# Critical engagement on the selection and reporting of biometeorological indices

**DOI:** 10.1007/s00484-026-03328-9

**Published:** 2026-09-22

**Authors:** Jennifer M. Fitchett

**Affiliations:** School of Geography, Archaeology and Environmental Studies, University of the Witwatersrand, Witwatersrand, South Africa

**Keywords:** Meteorological data, Data availability, Pre-evaluation, Ground-validation

## Abstract

**Abstract:**

Over the past four decades, a plethora of biometeorological indices has emerged. These are enabling the quantification and spatiotemporal comparison of climatic suitabilities and risks for a wide range of activities across indoor and outdoor settings. The relative mathematical simplicity of many of these indices, and the increasing availability of high-quality gridded data, mean that human biometeorological conditions can be computed rapidly and at scale. This is reflected in the literature, with considerable increases in quantitative biometeorological studies over the same period. However, with this computational ease comes a risk of the inadvertent output of spurious results; if the indices have not been carefully selected for their appropriate use for a given location, set of activities, and inherent assumptions. Where these biometeorological index outputs are used in informing policy, which ideally they should be, this risk of misinformation feeding maladaptation needs to be carefully mitigated through a thorough assessment of index applicability, suitability and validity. This paper sets out a conceptual selection framework that is guided by these three variables, following application of this approach in a range of settings, advocating for careful pre-assessment evaluation of indices in their selection.

**Clinical trial registration:**

Not applicable.

## Introduction

Biometeorological indices use weighted classifications of input meteorological variables to provide quantified and categorized scores, with associated qualitative evaluations of climatic suitability or risk for a particular activity. For example, in the case of tourism, there are a range of indices that use different weightings of a combination of thermal comfort, sunshine hours or cloud cover, rainfall, and wind to determine the level of climatic suitability of a destination for a given touristic attraction (Dubois et al. 2016; Prinsloo and Fitchett 2025). In the domain of thermal comfort, there are over 165 indices spanning a range of indoor and outdoor settings, and a number of different types of activities (de Freitas and Grigorieva 2017). As Grundstein and Vanos (2021) indicate, there is no ‘Swiss Army Knife’ of thermal indices, even in the more narrow domain of heat stress in sport, and Simpson (2024) illustrates the risks of applying the incorrect index to a given question in this sub-field.

Biometeorological indices have been developed to be widely-usable within this applied field. The majority have relatively simplistic mathematical operations, with the input of categorized meteorological data (McGregor 2012; Dubois et al. 2016), and output scores are classified descriptively, usually with reference to levels of suitability for a specific activity. With increased access to ground-based meteorological data, and the emergence of high-quality remote sensing and gridded datasets, input meteorological data are widely available. As a result, indices that rely purely on meteorological data as the input can, and have, been calculated across a wide range of spatio-temporal zones globally (e.g. Ma et al. 2021). This has allowed for the expansion of biometeorological research into under-represented geographical regions, which in turn can enable direct comparison between disparate environments. There remain challenges with regards to access to health datasets required in health biometeorology.

The use of indices distal to where they have been developed, although often now more feasible in terms of the data inputs, requires a considered approach (Fitchett and Meyer 2023). The process of index selection is not consistently reported in academic outputs, and recent debates suggest it is not always part of the study design (Dubois et al. 2016; Mushawemhuka 2021; Mnguni and Fitchett 2025; Prinsloo and Fitchett 2025). The ease of calculation and widespread use of the indices create an environment in which output scores and associated classifications may not represent human experiences in situ (Dubois et al. 2016). This poses a risk where the outputs of an index which may not be suitable to a given location are used in guiding climate adaptation particularly for vulnerable persons, and emerging industries (Scott et al. 2024).

Cognisant of this risk of maladaptation, this paper formally proposes a conceptual framework for the selection of biometeorological indices. This foregrounds the importance of explicitly reflecting on the applicability, validity, and suitability of an index when using it to answer a question of biometeorological investigation.

## Proposing a conceptual selection framework: applicability, validity and suitability

This conceptual framework draws from research conducted in southern Africa over the past decade using biometeorological indices (Saarinen et al. 2022). Located in the opposite hemisphere, and > 8000 km from the locations where these indices were developed, refined and tested, this has always required an implicit critical reflection on appropriate selection of methods to meaningfully represent human experience. This process became more explicit with the emergence of newer more activity-specific indices, which incorporated assumptions such as the ubiquity of air-conditioning which do not hold true in all socio-economic environments (Mushawemhuka 2021; Mnguni and Fitchett 2025). It is also the case where the assumptions of an index do not apply to the activity for which it has been applied, such as the case of the use of the Universal Thermal Climate Index (UTCI) in calculating the heat stress risk for the Comrades Marathon (Havenga et al. 2022). In this instance, the UTCI explicitly assumes no more exertion than a brisk walk, and clothing appropriate to the season, neither of which hold true for this ultramarathon that takes place in mid-winter (Simpson 2024). The risk herein is that the output scores and their interpretations could mislead adaptation strategies, particularly as evidenced by Mushawemhuka (2021), which can result in an over-estimation of the climatic suitability calculated for a given activity – whether tourism or running in these two examples, or any other activity for which a biometeorological index is used (Mnguni and Fitchett 2025).

**Fig. 1:**
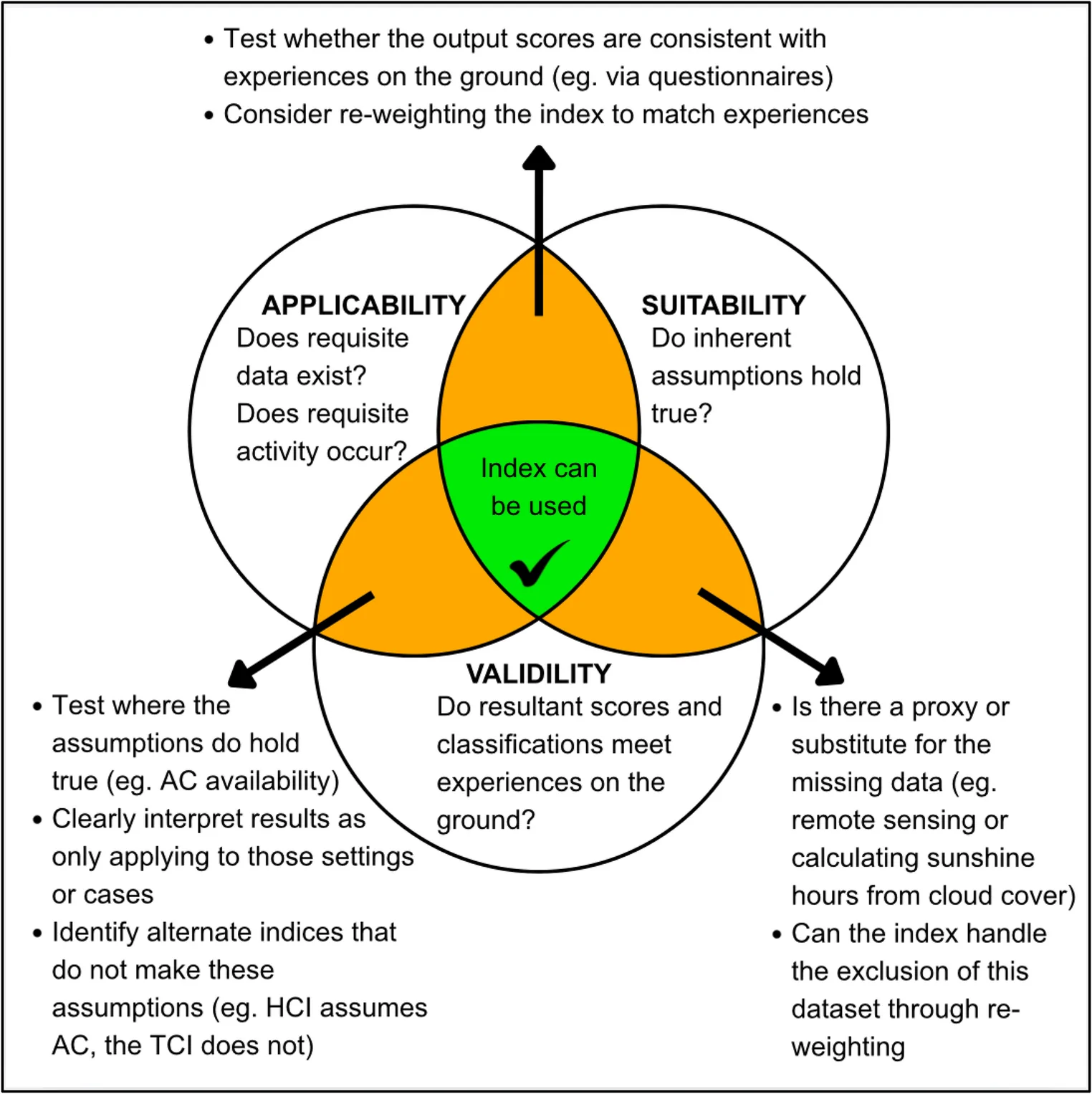
Theoretical framework for the critical selection of biometeorological indices. The green region in the center indicates cases where the index is found to be applicable, suitable, and valid, and can be used as is. The orange zones indicate an overlap of only two of the three components of index usability, requiring adaptation to address the shortfalls in the third, through the mechanisms indicated by the text for that region, indicated by the arrow from that segment

The theoretical framework for index selection proposed here includes the critical evaluation of the applicability, suitability, and validity of an index (Fig. 1) prior to implementation, recorded as part of the methodological design explicitly stated in any publication, and carefully considered in the interpretation of the output results.

Applicability refers in the first instance to whether the required meteorological data are measured for the location of interest. With increasing access to free gridded data products, biometeorological indices that rely only on meteorological indices can be applied for any location of interest, and has led to global outputs on index calculations (e.g. Ma et al. 2021). However, these gridded products are of a lower accuracy in smaller and mountainous regions (Prinsloo and Fitchett 2025), and should be compared to in situ data to determine local accuracy. Exploring the availability of locally measured high-quality data is therefore important in determining the applicability of an index to a given location. If locally measured data are not available, the second stage of evaluating applicability is in determining whether it is possible to substitute proxies for data that are not available (Noome and Fitchett 2019; Fitchett et al. 2016), or remove the missing variables while maintaining the integrity of the index (Fitchett et al. 2016). A key example here is sunshine hour data, which are key to the aesthetic component of many tourism climate indices, and for mental health analyses. For many countries, such data are only recorded for a small proportion of meteorological stations (Fitchett and Meyer 2023). Effective work-arounds have included the use of remote sensing data coupled with the time of sunrise and sunset to estimate sunshine hours in the eastern Lesotho Highlands (Noome and Fitchett 2019), and where sunshine hours remain at the maximum classification throughout the year, the re-weighting of indices to exclude this variable while retaining statistically indistinguishable resultant scores and classifications for stations that do have sunshine hour data (Fitchett et al. 2016). The estimation of sunshine hours from solar radiation data through a process of back-working a Food and Agricultural Organisation equation that calculates the inverse has been successful for the United States of America (Craig et al. 2023), but when tested for South Africa returns a large proportion of impossible values including negative sunshine hours, and daily sunshine hour totals that exceed the time from sunrise to sunset (Fitchett et al. 2025). This underscores the importance of testing any work-around in a local setting. Third, it considers whether the activity for which the index has been developed takes place in the location of interest, and forms part of the research question (Simpson 2024; Prinsloo and Fitchett 2025). While this third point may appear redundant, as one would not likely use a beach climate index inland, the Holiday Climate Index: Urban has increasingly been used, and indeed been encouraged for use through the peer review process, in rural environments spanning deserts to nature-based attractions in natural parks, when the older Tourism Climate Index is better suited to world tourism of this nature (Mushawemhuka et al. 2021). Where more than one activity does take place at a destination, and indices exist for the range of activities, a multi-index approach is more suitable than selecting one single index (Prinsloo and Fitchett 2025).

Suitability considers whether the inherent assumptions of the index hold true. The examples of the assumptions inherent in both the Holiday Climate Index and the Universal Thermal Climate Index raised earlier in this manuscript are key examples. Crucial to this is whether data can be obtained and evaluated to test these assumptions (Mnguni and Fitchett 2025). When considering the example of the Universal Thermal Climate Index, this may not need raw data, but rather is observational from the cohort of athletes taking part in the ultramarathon. When considering the assumption of air-conditioning availability, user profile analyses of accommodation establishments through booking websites can provide evidence of the proportion of listings for which this assumption does hold true, and their spatial distribution (Prinsloo and Fitchett 2024; Mnguni and Fitchett 2025; Kristensen and Fitchett 2026). If the assumptions do not hold universally true, the index outputs are still of value for the sub-population or sub-region for which they are true, but should be presented with these caveats (Prinsloo and Fitchett 2025). For example, the Holiday Climate Index outputs would remain representative of the climatic suitability for tourism for the subset of tourists who are staying in, and spending their evenings inside, air-conditioned accommodation establishments. Where assumptions do hold true at all, it is important to reflect this in the interpretation of the results presented (Simpson 2024; Mnguni and Fitchett 2025).

Validity refers to whether the output results and their classifications are consistent with human experience for the region and socioeconomic demographics of interest. This is explored through comparing human experience to the index outputs in the given location (Fitchett and Hoogendoorn 2019; Kganane and Fitchett 2025). While many indices are developed using data representing the human experience, including questionnaires and occupancy data, if these are developed in Europe or the United States of America one cannot assume that they would hold true in southern Africa (Fitchett and Meyer 2023; Kganane and Fitchett 2025), or indeed in South America, China or Australia. Crucial is whether the meteorological variables that are included in a given index are the same factors which influence climatic suitability for the given activity (Fitchett and Hoogendoorn 2019; Fitchett and Meyer 2023). Second is whether the weightings of the input variables align with their level of importance for the activity at the given location (Fitchett and Meyer 2023; Kganane and Fitchett 2025). This can be tested through questionnaires with local tourists (Fitchett and Meyer 2023; Kganane and Fitchett 2025), or through a netnographic analysis of reviews posted online by tourists visiting the destination (Fitchett and Hoogendoorn 2019). The latter can be done increasingly more efficiently through the use of web-scraping (Mokgehle and Fitchett 2024), and provides an arguably more objective measure of climatic sensitivity as it removes the potential for any questions to trigger reflection specifically on climate (Fitchett and Hoogendoorn 2019). Although ideally an analysis of validity would be conducted for any given region of interest, where the climate zones and tourism population demographics remain similar, a verification for one country or region could be considered indicative of surrounding regions (Prinsloo and Fitchett 2025). It should not, however, be considered representative of a full continent, hemisphere, or similarly scaled geographic region, or one with a very different climate regime.

## Weighing up index refinement versus inter-study comparability

Ideally, an index should only be applied where it is found to be applicable, valid, and suitable to a given location and activity (indicated by the green region of Fig. 1). However, where no index meets all these criteria for a given setting where a biometeorological question would be of value to answer, further index development or alteration may be required (as in each of the orange regions of Fig. 1). This could include the substitution of data to a proxy thereof (Noome and Fitchett 2019; Fitchett et al. 2025), reweighting of the index to remove a variable (Fitchett et al. 2016), or re-weighting an index with all of the included variables to meet the infrastructural conditions or local preferences of tourists (Kganane and Fitchett 2025). Here, the key consideration is as to whether the level of development is of a sufficiently limited extent to allow for continued inter-study comparison of index outputs (Fitchett and Hoogendoorn 2019). Such comparisons between studies and regions are not inherently required, but where they are made it is important to consider the extent to which outputs compared are equivalent in their structure and input variables to ensure comparison of like-for-like. While the development of activity- and location-specific indices improves their level of accuracy in representing human-experience on the ground, an over-proliferation of new indices hinders the ability to compare relative suitability regionally, globally, and between activities.

## Conclusion

This paper formalizes a conceptual framework to considerations in the selection and reporting of biometeorological indices, to ensure that the quality of data inputted is aligned with the accuracy of the output score and classification, compared to the human experience. The explicit critical analysis of the applicability, suitability, and validity of an index should comprise the starting point of any study using biometeorological indices, as a component of the methodological design. To encourage this approach, and for scientifically robust reporting, it is valuable where possible to detail this in the methods section of such a paper. This both improves the accuracy of the interpretation of output data, and allows for the comparability between studies, which at its core requires clarity as to the similarity of not only methods but data sources, substitutions and assumptions made.
